# Chrononutrition in the management of diabetes

**DOI:** 10.1038/s41387-020-0109-6

**Published:** 2020-02-19

**Authors:** Christiani Jeyakumar Henry, Bhupinder Kaur, Rina Yu Chin Quek

**Affiliations:** 1 Clinical Nutrition Research Centre, Singapore Institute for Clinical Sciences, 14 Medical Drive, #07-02, Singapore, 117599 Singapore; 2 National University of Singapore, Department of Biochemistry, 8 Medical Drive, Singapore, 117596 Singapore

**Keywords:** Type 2 diabetes, Nutrition

## Abstract

Circadian rhythms are 24-h cycles regulated by endogeneous molecular oscillators called the circadian clock. The effects of diet on circadian rhythmicity clearly involves a relationship between factors such as meal timings and nutrients, known as chrononutrition. Chrononutrition is influenced by an individual’s “chronotype”, whereby “evening chronotypes” or also termed “later chronotype” who are biologically driven to consume foods later in the day. Research in this area has suggested that time of day is indicative of having an influence on the postprandial glucose response to a meal, therefore having a major effect on type 2 diabetes. Cross-sectional and experimental studies have shown the benefits of consuming meals early in the day than in the evening on postprandial glycaemia. Modifying the macronutrient composition of night meals, by increasing protein and fat content, has shown to be a simple strategy to improve postprandial glycaemia. Low glycaemic index (GI) foods eaten in the morning improves glycaemic response to a greater effect than when consumed at night. Timing of fat and protein (including amino acids) co-ingested with carbohydrate foods, such as bread and rice, can reduce glycaemic response. The order of food presentation also has considerable potential in reducing postprandial blood glucose (consuming vegetables first, followed by meat and then lastly rice). These practical recommendations could be considered as strategies to improve glycaemic control, rather than focusing on the nutritional value of a meal alone, to optimize dietary patterns of diabetics. It is necessary to further elucidate this fascinating area of research to understand the circadian system and its implications on nutrition that may ultimately reduce the burden of type 2 diabetes.

## Introduction

Diabetes mellitus remains a leading chronic disease in the world with the number of diabetics quadrupling in the past three decades^1^. The International Diabetes Federation (IDF) estimated that 415 million adults had diabetes in 2015, and by 2040, it is projected to reach 642 million^2^. In concert pharmacological interventions, dietary interventions remain the cornerstone of diabetes prevention and management. The key therapeutic approach to reducing the incidence and severity of type 2 diabetes focuses on the nature and quality of nutrients consumed.

Circadian rhythms are 24-h cycles regulated by endogeneous molecular oscillators called the circadian clock^3^. The mammalian circadian system comprises of various individual tissue-specific clocks. This circadian clock prepares the body for events that take place throughout the day. These include physiological parameters such as hormone secretion, heartbeat, renal blood flow, the sleep-wake cycle and body temperature fluctuations^4^. The circadian clock is located in the suprachiasmatic nuclei (SCN) and is a central regulator of the peripheral clock system. It plays an important role in regulating several physiological processes which synchronizes to the central 24-h circadian rhythm^5^. When the SCN is destroyed, circadian rhythms of the sleep cycle and the release of various hormones diminishes. The SCN contains several types of peptide-synthesizing neurons that are essential for the entrainment and shift of circadian rhythms with the most prominent being the somatostatine neurons, vasoactive intestinal peptide and arginine vasopressin^6^. Ensuring circadian rhythmicity is crucial in influencing and regulating metabolic processes by regulating the expression and/or activity of enzymes involved in glucose metabolism. In recent years, a growing body of evidence is emerging that the circadian clock system can interact with nutrients to influence bodily functions. This relatively new field is described as “chrononutrition”^7,8^. In modern society, numerous occupations and the high prevalence of insomnia lead to lifestyles that are not aligned with their circadian clock^9^. The lack of alignment with the circadian clock has been reported to influence food intake, glucose metabolism, weight regulation and obesity^10–12^.

Although animal and cell models have been the experimental focus in delineating the impact of the circadian clock on physiological and nutrition, an emerging body of evidence is also being generated from human studies. The present review (although focusing predominantly on healthy subjects) aims to collate information that is also relevant to diabetics in relation to their meal timings and nutrient intake influencing glycaemic control.

## Circadian rhythm

### Circadian rhythm and glucose metabolism

**Fig. 1:**
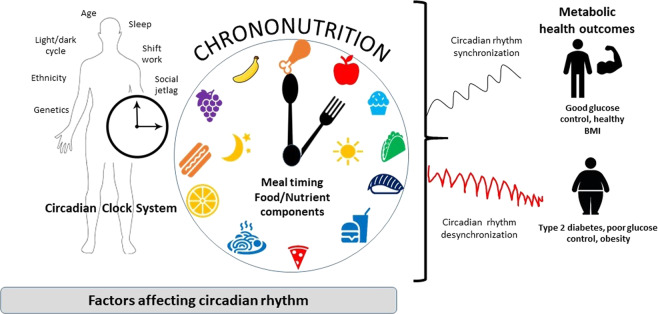
A schematic representation outlining the factors affecting the circadian clock system. Meal timing and dietary components (chrononutrition) play an important role in regulating circadian clocks, to enhance metabolic health and reduce the risk of type 2 diabetes.

From a chronobiological point, glucose metabolism in humans follow a circadian rhythm through diurnal variation of glucose tolerance that typically peaks during day-light hours, when food consumption usually happens and reduces when it comes to night-dark hours when fasting usually occurs^13^. Several hormones involved in glucose metabolism, such as insulin and cortisol, exhibit circadian oscillation^14,15^. For example, experiments in rodents have shown the importance of the circadian system in glucose metabolism with changes in insulin sensitivity and insulin secretion patterns inducing highly rhythmic changes, thus affecting blood glucose levels^16–18^. Therefore, insulin secretion and sensitivity are closely regulated by circadian control and have strong effects on glucose metabolism. Unusual meal timings can cause glucose intolerance by affecting the phase relationship between the central circadian pacemaker and peripheral oscillators in cells of the liver and pancreas in rodents^19^. Similarly in humans, timed meal intake is also driven by the SCN, play a role in synchronization of circadian rhythms in peripheral tissues, thereby affecting glucose metabolism^7,8,20^. The effects of diet on circadian rhythmicity clearly involves a relationship between factors such as meal timings and nutrients (chrononutrition), that can contribute to circadian perturbance and influence the manifestation of metabolic disorders such as type 2 diabetes (Fig. 1).

## Meal timings and glucose metabolism

### Skipping breakfast and late meal time

Since the middle of the 20th century, eating patterns have shifted towards later eating times with over one-third of the caloric intake consumed after 6 pm^21^. This late-eating pattern, common in the modern lifestyle today, may lead to circadian misalignment and therefore exert a negative impact on glucose control. Circadian misalignment is also increasingly found in night shift workers. As human beings are diurnal species and generally sleep at night, shift workers are prone to developing sleep disturbances when the relationship between the light-dark phase and food intake is desynchronized. Considerable epidemiological evidences have shown that the disruption of the biological circadian clock is negatively associated with various metabolic diseases such as obesity, cardiovascular disease, gastrointestinal problems and diabetes^4,22^. Shift workers tend to have a higher basal metabolic index (BMI) compared with day workers^23,24^. Several studies displayed a negative relationship between shift work and metabolism. Shift workers exhibited a lowered glucose and lipid tolerance following a change from day to night shift work^25–27^. Insulin resistance was also demonstrated to be more recurrent in shift workers who were 50 years or younger^28^. Another study equated the risk of shift work to the risk of smoking one pack of cigarettes a day in spite of controlling for other confounders and risk factors^29^. The complexity of the phenotypic expression of obesity is influenced by numerous factors such as stress, social rhythm, altered patterns of transcriptional genes, altered glucose and lipid homoeostasis, disruption of the central and peripheral oscillators and a decreased thermogenic response during night eating^30^.

Emerging evidence also suggests that chrononutrition is influenced by an individual’s “chronotype”. Chronotype is a behaviour manifestation of an individual’s internal circadian clock system, whereby they can be classified to have a preference for the morning or evening^9^. Individuals with an “evening chronotype” or also termed “later chronotype” are biologically driven to consume foods later in the day^31^.

There has been a relatively strong association between breakfast skipping and insulin resistance or type 2 diabetes shown in some studies. In a 16-year follow-up cohort study, using the Cox proportional hazards analysis, it was shown that after adjusting for known risk factors of T2D including BMI, US men who skipped breakfast had a 21% higher risk of developing T2D compared with men who consumed breakfast^32^. In another randomised crossover trial, 10 women underwent a 2-week intervention with a 2-week washout period of either skipping or consuming breakfast. The study demonstrated that omitting breakfast led to a significantly higher energy intake, fasting total and LDL cholesterol, and a significantly lower postprandial insulin sensitivity^33^. Individuals who have a preference for late-night dinner consumption (later chronotypes) may have a tendency to skip breakfast the following morning^34^. This may be due to insufficient time to eat during the day or due to reduced hunger in the morning. A large cross-sectional study in healthy Japanese individuals, after adjusting for body mass index (BMI), showed that breakfast skipping was not the sole cause of hyperglycaemia but also a result of late-night dinner consumption^35^. Kobayashi et al. reported that healthy males who skipped breakfast and then consumed large meals at lunch and dinner, had greater postprandial glucose, especially after dinner^36^. An acute study in type 2 diabetics, who were classified as later chronotypes and skipped breakfast, had poorer glycaemic control as indicated by their significantly higher HbA1c values^31^. A cross-sectional study by Sakai et al. reported that there was an independent association of having a late-night dinner and skipping breakfast with poor glycaemic control in individuals with type 2 diabetes^37^.

The omission of breakfast further compounded by late-night meal consumption, may delay circadian rhythms. These individuals do not consume breakfast due to lack of appetite signalling brought about by the disruption of biological clocks^38^. Due to inappropriate time of feeding, the misalignment of the circadian clock leads to worsening of glycaemic control and an increased risk of developing type 2 diabetes.

However, there is another body of evidence suggesting that breakfast is not the most important meal of the day, rather, it is ‘just another meal’ as there has yet to be an established causal relationship between skipping breakfast and its negative metabolic implications. The authors highlighted that in most breakfast studies comparing the effects of consuming or skipping breakfast, the duration of overnight fast was not accounted for^39^. This was an important confounder that should be considered. For example, two breakfast consumers both had their last meals at 2200 h the night before, one ate breakfast the next morning at 0600 h compared with one that consumed breakfast only at 1000 h, the difference in duration of overnight fast could potentially be the cause for a prominent difference metabolically. Conversely, regardless of time of meal consumed, similar durations of overnight fast may have resulted in similar metabolic profiles.

Likewise, despite routine evidences indicating that skipping breakfast resulted in an increased BMI and an overcompensation of energy consumed later during the day, no causality has been established^40,41^. Zilberter and Zilberter also discussed indirect potential benefits of omitting breakfast in relation to intermittent fasting where the suppression of appetite could lead to voluntary caloric reduction and caloric restriction may have profound metabolic effects on human health^39,42,43^.

### Meal intakes timing of ingestion: morning versus dinner

Evidence suggests that meal ingestion in the morning and late evening (time of day) influence glucose metabolism in humans. Earlier studies, using mixed meals or glucose infusion, have reported circadian responses of reduced glucose tolerance and insulin sensitivity in healthy participants for the evening rather than in the morning^44–47^. An acute study by Sato et al. examined the effects of a late evening meal on diurnal variation of blood glucose in healthy individuals, assessed by continuous glucose monitoring (CGMS™)^48^. There was an increase in blood glucose after the late evening meal which shifted towards later at night, with peaking of blood glucose observed during sleep^48^. Late evening meals may cause postprandial hyperglycaemia with this decrease in glucose tolerance from morning towards the night.

A cohort study was the first to show the relationship between late-night dinner consumption and glycaemic control in type 2 diabetics, whereby having a late dinner meal after 8 pm was independently associated with an increase in HbA1c^37^. In some acute trials, both healthy individuals^49,50^ and type 2 diabetics^51^ showed significantly higher blood glucose and insulin values after night-time meals. These studies deduce that the disruption of the circadian rhythm led to the exacerbation of the physiological nocturnal decrease of glucose tolerance. Peter et al. showed that type 2 diabetic subjects who ate three identical meals had glucose excursions that were higher in the morning than in the evening^52^. There was increased glucose tolerance in response to the first and third meals of the day, irrespective of glycaemic control. There was also a change in circadian variation in insulin sensitivity in type 2 diabetics^52^. Type 2 diabetics exhibit a different daily circadian pattern from healthy individuals, with increased insulin sensitivity towards the night, and higher glucose excursions in the morning than in the evening.

Methods to improve glycaemic control at dinner has been reported by some authors. Dinner that was divided into two smaller meals reduced post-meal glycaemic excursions, due to the “second-meal effect” phenomenon, by enhancing β-cell responsiveness^53^ at the second dinner meal induced by the first meal^49,51^. It was also suggested that if meal size and carbohydrate quantities were smaller, postprandial glucose can be ameliorated in both healthy and type 2 diabetics.

In summary, time of day is indicative of having an influence on the postprandial glucose response to a meal. There is a defined circadian pattern for postprandial glycaemia for similar meals consumed either in the evening or morning. These studies illustrate that glucose metabolism is not only affected by what and how much you eat alone, but also when the meal is consumed. However, more data from well-designed epidemiological studies is necessary to prove causality.

## Time of nutrient intake and glucose metabolism

It is evident from literature that there is an obvious circadian pattern to have a higher postprandial glucose response to meals at night than in the morning, in healthy individuals. With the increasing trend of people shifting towards a late-eating pattern (later chronotype) due to several lifestyle factors and changes, understanding how diet can be manipulated is crucial to ensure circadian synchronization so as to improve glycaemic control. Meal composition, in addition to meal timing, also appears to influence glucose levels.

### Calories

Current evidence suggests that the time of day in which the amount of calories is consumed, can affect glycaemic control. Some animal studies have shown that there is an impairment of peripheral clock gene expressions due to skipping breakfast or reduced food intake in the first meal of the day, along with high-caloric dinners (despite no differences in daily total caloric intake), resulting in higher daily glucose excursions^54,55^.

A cohort study reported that when most of the day’s calorie requirements were consumed at dinner, there was a 2-fold greater incidence of diabetes in older men and women^56^. In a randomized, parallel-arm designed study, the effect of consuming a high calorie meal in the morning (i.e. breakfast) versus a high calorie meal in the evening, was assessed in overweight and obese women (with metabolic syndrome)^57^. The group who consumed more calories at breakfast saw a greater reduction in fasting blood glucose and insulin when compared with consuming more calories at dinner^57^. This was taking into account the fact that there was a similar daily calorie intake for both arms. Furthermore, a reduction in glycaemic and insulinemic responses for oral glucose tolerance test (OGTT) after a high calorie breakfast compared with a high calorie dinner was also observed^57^. In a crossover study, type 2 diabetic participants who were given a high-caloric breakfast/low caloric dinner (contrasting arm was a low caloric breakfast/high-caloric dinner), was associated with reduction in postprandial hyperglycaemia, increase in insulin and glucagon-like peptide-1 (GLP-1) for the whole day^58^. The results of this study by Jakubowicz et al. demonstrated the diurnal variation of glycaemic control in type 2 diabetics^58^. The amount of calories consumed at breakfast or dinner seemed to have an influence on the daily rhythm of postprandial glycaemic excursions and insulin levels.

For most people, it would be unrealistic to avoid eating at night. With night-time eating being associated with poor glucose control and the increased risk of type 2 diabetes, manipulating meal composition of late meals or reducing the portion size may be crucial strategies to positively impact on postprandial glucose.

### Carbohydrates

The quantity, quality type, and rate of digestion of dietary carbohydrates are the primary determinants of postprandial glucose levels and insulin response^59,60^. Therefore, they all play an important role in the diet we consume, when it comes to circadian rhythmicity and glycaemic control. Epidemiological studies have shown that the time of consumption of carbohydrate-rich meals, if at the start of the day, has protective benefits against the development of diabetes^61,62^. Acute trials have reported that, specifically nocturnal consumption of carbohydrates, an increased absorption of dietary cabohydrates resulted in a higher postprandial glucose profile the following morning^63,64^.

#### Glycemic index (GI) and meal timings

The glycemic index (GI) is defined as the blood glucose raising potential of carbohydrate foods^65^. Low GI carbohydrates have shown to be beneficial as they have a lower impact on blood glucose concentrations and protect against hypoglycaemia^66^. There is good evidence to suggest that it also avoids large fluctuations in blood glucose levels^67^.

Some intervention studies have aimed to establish if varying the GI and the time at which meals are consumed impacts on postprandial glucose and insulin responses. A four-way, randomized crossover study in healthy individuals, by Morgan et al. compared the glycaemic effects of varying the GI and glycaemic load (GL) (GL = GI × carbohydrate content) and the timing of meal consumption, with most of the energy consumed either for breakfast or for dinner^68^. In their first observation, higher GL meals consumed in the evening led to higher glucose and insulin response compared with consuming the same meal in the morning. In the second part of the study, a high GI diet given in the evening, produced an even more pronounced effect on glucose and insulin^68^. These results confirmed that the quality and quantity of carbohydrates, i.e. the GI and GL in addition to the time at which the meal is consumed, influences glycaemic control and insulin secretion. An additional randomised crossover study in healthy subjects investigated the effect of low and high GI meals on glucose levels, when given in the earlier or latter part of the day^69^. Postprandial glucose response in the evening was greater even after the low GI meal^69^. These results suggest that low GI foods, even if they were consumed in the night, was less efficient in glucose control. Low GI foods were more effective in glucose control in the morning. This could possibly be explained by the changes in insulin sensitivity which has been reported to decrease during the day^70^. Furthermore, an additional influence is due to hormones such as glucagon and cortisol which are affected by circadian rhythms^71^, and in turn influence insulin secretion and glycaemic response. A recent intervention investigated the timing of low GI meals in the morning (0800 h), evening (2000h) and midnight (0000 h)^72^. The low GI meals consumed in the evening and midnight resulted in higher glucose excursions with concomitant higher insulin levels, compared with the morning^72^. Collectively, these studies have shown that having a low GI meal, irrespective of meal size, improved glycaemic response in the morning but had little impact at night. This temporal difference has been associated with the effect that the endogenous circadian rhythm has on glucose metabolism^73^.

### Fats

Epidemiological studies has reported that consumption of more carbohydrates than fats in the morning prevents the development of diabetes and metabolic syndrome^61,62^. The effect of manipulating fats, as well as carbohydrates, in day and night meals on postprandial glycaemic response has been undertaken in a few experimental studies. A randomized crossover trial in healthy men compared whether consuming a high carbohydrate diet or a high fat diet during different timings in a 24 h period, would produce different plasma glucose responses^74^. A more rapid rise in plasma glucose was observed with the high carbohydrate diet compared with the high fat diet. There was also a circadian pattern in plasma glucose concentration, with the circadian effect coming from the high fat diet consumption^74^. A recent randomised crossover trial comparing two isocaloric meals, differing in total sugar and saturated fat, was undertaken during a simulated night shift work in overweight males with high fasting lipids^75^. Although this study resulted in no significant changes in circadian gene expressions, modifying a meal by reducing saturated fat and sugar for a dinner meal was associated with improved glucose response^75^. Whilst the quality of fat ingested is known to influence metabolism, there is a lack of consistent information on the degree of saturation and chain length of fatty acids influencing postprandial glycaemia and lipidemia. This further highlights the need to investigate the chronobiology of dietary fat intake on glucose homoeostasis.

### Proteins

A recent crossover study on healthy participants examined if a high protein meal could attenuate postprandial glucose in the morning and at night^76^. The effect of a high protein meal showed a significant modulation of the glucose response at night, with a significantly lower incremental area under the curve (iAUC) compared with a standard meal^76^. There were no differences in iAUC glucose for morning between the high protein test meal and standard meals. In addition, there were no differences noted for insulin responses between meal type in the morning or night^76^. These findings suggest that increasing the amount of proteins in a meal can reduce postprandial glucose at night. This can be beneficial for people who are late chronotypes or late-night eaters, who are more predisposed to glycaemic excursions and therefore reduce the risk of hyperglycaemia. The glucose-attenuation property of dietary protein seems to be also influenced by the timing of consumption. However, there are limited studies to support this and more needs to be done on the glycaemic and insulinemic impact of protein in meals, in accordance to timing of day.

## Timing fat and protein foods to lower glycaemic response of carbohydrate meals

The consumption of high GI starchy foods, such as white rice and white bread, have been implicated in the development of type 2 diabetes. Therefore, investigating the timing of fat and protein (including amino acids) co-ingestion with carbohydrate foods remains a novel food-based intervention to reduce glycaemic response (GR).

The ingestion of fat in the form of olive oil, half an hour before a potato meal, was found to attenuate postprandial glucose and insulin in type 2 diabetics^77^. Milk protein given as a preload prior to consuming bread rather than coingesting both foods, was found to significantly lower postprandial glycaemia and insulinemia^78^. Essence of chicken (EOC), a chicken meat extract which is a rich source of peptides and amino acids, is commonly consumed in Asian countries. There has been a long debate on the timing at which EOC should be consumed for maximum health benefits. One study showed that the co-ingestion of EOC with white bread was a simple strategy to reduce the glycaemic response of bread^79^. More interestingly, the same group found that the ingestion of EOC 15 min prior to the consumption of white rice elicited the greatest reduction in glycaemia^80^. The results suggest that timing of ingestion plays a significant role in insulin secretion which in turn impacts on glucose homoeostasis. A most recent study by the team also examined meal sequence as being an important regulator of postprandial glucose. Consumption of vegetables, followed by meat and then lastly rice, was the best sequence to attenuate glycaemic response without an increased demand for insulin in healthy adults^81^. The sequence of presenting food and their timing of consumption has a great impact in modulating glycaemic response.

In summary, it is now recognized that there is an increased glycaemic excursion and reduced insulin sensitivity when meals are consumed at night than during the day. These studies highlight the interaction of meal timing and nutrient composition (carbohydrate, fat and protein) on glucose metabolism. The application of these observations also include the timing at which fat and protein foods are consumed during a carbohydrate-rich meal. A more recent observation is how the sequence of food presentation within a meal can also influence glycaemic and insulinemic response. Collectively, these observations are easily transferable as public health advocacy to communities that have a high prevalence of type 2 diabetes (pre-diabetes) and are dependent on a high carbohydrate diet.

## Timing the consumption of food components for glycaemic control

Some food components have been identified as having the ability to modulate circadian clocks and impact glycaemic control, with many such studies being conducted in animals^14^. A few human studies have reported the role of food components when consumed at specific timings. Green tea polyphenols, such as catechins, have shown to be beneficial in decreasing fasting and postprandial glucose^82,83^. Most recently, it was demonstrated for the first time that ingesting catechin-rich green tea in the evening was able to reduce postprandial plasma glucose concentrations compared with the placebo tea given at the same time^84^. Epidemiological studies have reported the protective effect of coffee consumption on the development of diabetes^85,86^. The effects of coffee on postprandial glucose differs when consumed at different times of the day, indicating that timing of coffee intake may have a preventative effect on type 2 diabetes risk. A prospective cohort study reported an association between both caffeinated coffee and decaffeinated coffee and reduced risk of type 2 diabetes, but only when coffee was consumed at lunch time^87^. In a randomized crossover trial, caffeinated coffee consumed in the morning had a higher postprandial glucose and insulin response to a later meal^88^. These small but important findings suggest that the circadian clocks can be affected by food components, depending on the time they are consumed. This is an approach for maintaining glucose homoeostasis that merits further investigation.

## Conclusion

Whilst chrononutrition is an advancing science, there still remains much to be learnt about the nature and timing of diet provision in regulating glucose homoeostasis. This review has demonstrated that the choice of food alone does not dictate glycaemic response. The emerging field of chrononutrition indicates that the timing and order of food presentation within and between meals could also significantly influence postprandial glycaemia. There still remains much to be learnt. We hope that this paper will stimulate further research that will enable us to translate how chronobiology may be effectively used in communities around the world that are confronted with the burgeoning prevalence of type 2 diabetes.

### Key messages

An overall summary of key studies reporting the effects of meal timings and dietary factors on glycaemic control is shown in Table 1.

- Meal timing has a major effect on type 2 diabetes. It is therefore important to consider the timing of meal consumption rather than focus on the nutritional value of a meal alone.
- Eating a carbohydrate-rich meal at night results in increased postprandial glycaemia compared with an identical meal in the morning. Therefore, modifying the macronutrient composition of meals, by increasing protein and fat content, can be a simple strategy to improve glycaemia for meals consumed at that night.
- The benefits of consuming meals early in the day should be encouraged in diabetics.
- Eating low GI foods in the morning improves glycaemic response to a greater effect than at night.
- Timing of fat and protein (including amino acids) consumption with carbohydrate foods, such as bread and rice, can reduce the glycaemic response.
- The order of food presentation considerably influences the glycaemic response. For a rice-based meal, following the sequence of consuming vegetables first, followed by meat and then lastly rice, has great potential of reducing the postprandial blood glucose.

**Table 1:** Summary of studies reporting the effects of meal timings and dietary factors on glycaemic control.

Authors	Year of publication	Participants	Main finding	Ref. no.
Sakai et al.^37^	2018	Men and women Type 2 diabetics	Late-night dinner independently associated with poor glycaemic control	^37^
Kajiyama et al.^49^	2018	Young women Healthy	Late-night dinners increased postprandial hyperglycaemia Consuming dinner dividedly ameliorate postprandial glucose levels	^49^
Imai et al.^51^	2017	Men and women Type 2 diabetics	Late-night dinners increased postprandial hyperglycaemia	^51^
Van Cauter et al.^46^	1992	Men and women Healthy	For identical mixed meals, total and 2-h AUC were 25–50% greater in the evening than in the morning	^46^
Jakubowicz et al.^57^	2013	Women Overweight and obese metabolic syndrome (BMI: 32 kg/m^2^)	High-energy breakfast and reduced-energy dinner significantly reduces postprandial glycaemia in obese non-diabetic individuals	^57^
Jakubowicz et al.^53^	2015	Men and women Type 2 diabetics	High-energy breakfast and reduced-energy dinner significantly lowered postprandial glycaemia	^58^
Jakubowicz et al.^53^	2015	Type 2 diabetics	Skipping breakfast associated with a worsened glycaemic response after lunch and dinner in type 2 diabetics	^53^
Bo et al.^56^	2014	Men and women (45–64 years) Exclusion: obesity and/or diabetes mellitus at baseline	Individuals in highest tertile of dinner, % daily caloric intake showed increased risk of incident type 2 diabetes	^56^
Bo et al.^89^	2015	Men and women Healthy	Delayed and larger rises in glucose and insulin concentration after evening meals	^89^
Bandin et al.^19^	2015	Women Healthy	Late eating of lunch showed increased postprandial glucose than early-eating	^19^
Tsuchida et al.^64^	2013	Women Healthy	Late supper increased postprandial serum glucose profiles the following morning	^64^
Al-Naimi et al.^50^	2004	Men Healthy	Meals and snacks eaten during simulated night shift work had higher postprandial glucose Glucose tolerance impaired after first night-time meal, with no differences observed following second meal	^50^
Sato et al.^48^	2011	Men and women Healthy	Increase in postprandial glucose after late evening meal which carried over to breakfast	^48^
Reutrakul et al.^31^	2014	Women Type 2 diabetics	Breakfast skipping significantly associated with higher HbA1C values	^31^
Peter et al.^52^	2010	Men and women Type 2 diabetics	Morning glucose excursions higher than in the evening Decreased glucose tolerance for first and third meal of the day	^52^
Kobayashi et al.^36^	2014	Men Healthy	Breakfast skipping, and big meals at lunch and dinner, had greater postprandial glucose, especially after dinner	^36^
Takahashi et al.^90^	2018	Men Healthy	Higher postprandial glucose for evening meal than morning meal	^90^
Morgan et al.^68^	2012	Men and women Healthy	Glucose levels higher with late consumption of high GI meals compared with low GI meals Insulin sensitivity worsens with high-energy meals consumed in evening	^68^
Gibbs et al.^69^	2014	Men and women Healthy	Postprandial glucose AUC showed effect with time of day after both low GI and high GI meals Higher glycaemic responses in the evening for low GI meal	^69^
Leung et al.^72^	2019	Men and women Healthy	Low GI meals at night contribute to higher glucose excursions and greater insulin levels compared with low GI meals in morning	^72^
Kessler et al.^63^	2017	Men Non-obese (normal and impaired glucose, fasting tolerance)	Increased absorption of dietary cabohydrates late night resulting in a higher postprandial glucose profile the following morning	^63^
Davis et al.^76^	2019	Men and women Healthy	High protein meal attenuates glucose excursions compared with a standard meal at night	^76^
Bonham et al.^75^	2019	Men Healthy	Modifying night meal by reducing saturated fat and sugar improved postprandial glucose	^75^
Holmbäck et al.^74^	2002	Men Healthy	High fat diet significant circadian pattern for plasma glucose compared with high carbohydrate diet	^74^
Gentilcore et al.^77^	2006	Type 2 diabetics	Olive oil consumed 30 min before a potato meal attenuated postprandial rise in glucose	^77^
Sun et al.^78^	2017	Men Healthy	Milk protein consumed before eating bread reduced GR	^78^
Sun et al.^79^	2015	Men Healthy	Essence of chicken co-ingested with white bread reduced GR compared with white bread alone	^79^
Soong et al.^80^	2015	Men Healthy	Essence of chicken co-ingested with white rice reduced GR compared with white rice alone	^80^
Takahashi et al.^84^	2019	Women Healthy	Green tea consumption beneficial in the evening to reduce postprandial glucose concentrations by 3% lower than placebo	^84^
Sartorelli et al.	2019	Women	Coffee and caffeine intake at lunch time inversely associated with the risk of diabetes	^87^
Lund et al.^26^	2001	Men and women Shift workers	Observed abnormal metabolic responses for meals consumed at night during, due to insulin resistance	^26^
Hampton et al.^27^	1996	Men and women Shift workers	Significant higher postprandial glucose and insulin responses at phase shift (body clock time 2230 h)	^27^
